# Loregic: A Method to Characterize the Cooperative Logic of Regulatory Factors

**DOI:** 10.1371/journal.pcbi.1004132

**Published:** 2015-04-17

**Authors:** Daifeng Wang, Koon-Kiu Yan, Cristina Sisu, Chao Cheng, Joel Rozowsky, William Meyerson, Mark B. Gerstein

**Affiliations:** 1 Program in Computational Biology and Bioinformatics, Yale University, New Haven, Connecticut, United States of America; 2 Department of Molecular Biophysics and Biochemistry, Yale University, New Haven, Connecticut, United States of America; 3 Department of Genetics, Geisel School of Medicine at Dartmouth, Hanover, New Hampshire, United States of America; 4 School of Medicine, Yale University, New Haven, Connecticut, United States of America; 5 Department of Computer Science, Yale University, New Haven, Connecticut, United States of America; Rutgers University, UNITED STATES

## Abstract

The topology of the gene-regulatory network has been extensively analyzed. Now, given the large amount of available functional genomic data, it is possible to go beyond this and systematically study regulatory circuits in terms of logic elements. To this end, we present Loregic, a computational method integrating gene expression and regulatory network data, to characterize the cooperativity of regulatory factors. Loregic uses all 16 possible two-input-one-output logic gates (e.g. AND or XOR) to describe triplets of two factors regulating a common target. We attempt to find the gate that best matches each triplet’s observed gene expression pattern across many conditions. We make Loregic available as a general-purpose tool (github.com/gersteinlab/loregic). We validate it with known yeast transcription-factor knockout experiments. Next, using human ENCODE ChIP-Seq and TCGA RNA-Seq data, we are able to demonstrate how Loregic characterizes complex circuits involving both proximally and distally regulating transcription factors (TFs) and also miRNAs. Furthermore, we show that MYC, a well-known oncogenic driving TF, can be modeled as acting independently from other TFs (e.g., using OR gates) but antagonistically with repressing miRNAs. Finally, we inter-relate Loregic’s gate logic with other aspects of regulation, such as indirect binding via protein-protein interactions, feed-forward loop motifs and global regulatory hierarchy.

## Author Summary

Gene expression is controlled by various gene regulatory factors. Those factors work cooperatively forming a complex regulatory circuit genome wide. Corruptions of regulatory cooperativity may lead to abnormal gene expression activity such as cancer. Traditional experimental methods, however, can only identify small-scale regulatory activity. Thus, to systematically understand the cooperativity between and among different types of regulatory factors, we need the efficient and systematic computational methods. Regulatory circuits have been suggested to behave analogously to the electronic circuits in which a wide variety of electronic elements work coordinately to function correctly. Recently, an increasing amount of next generation sequencing data provides a great resource to study regulatory activity. Thus, we developed a general-purpose computational method using logic-circuit models from electronics and applied it to a human leukemia dataset, identifying the genome-wide cooperativity of transcription factors and microRNAs.

This is a *PLOS Computational Biology* Methods paper

## Introduction

The rapidly increasing amount of high throughput sequencing data offers novel and diverse resources to probe molecular functions on a genome-wide scale. Integrating and mining these various large-scale datasets is both a central priority and a great challenge for the field of functional genomics and necessitates the development of specialized computational tools.

Gene expression is a complex process that achieves both spatial and temporal control through the coordinated action of multiple regulatory factors (RFs) [1–3]. These regulatory factors affecting gene expression take several forms, such as transcription factors (TFs), which directly or indirectly bind DNA at promoter and enhancer regions of their target genes, and non-coding RNAs (e.g. miRNAs) [4,5]. RFs can act as activators or repressors, but ultimately, the target gene expression is determined by combining the effects of multiple regulatory factors. As a large amount of genomic data has become available, it is possible to systematically study the genomic functions of various RFs and see how they interact with each other in order to regulate target gene expression.

In the past decade, an increasing number of experimental and computational studies have focused on analyzing links between RFs from various biological characteristics such as protein-protein interactions, sequence motifs in *cis*-regulatory modules of TF binding sites, co-associations of TFs in binding sites, and co-expressions of TF target genes [1,5–8]. However, to date, large-scale studies have generally been limited to identifying RF “wiring relationships” (e.g. co-binding, co-association), leaving untouched the cooperative patterns among RFs that drive the biological functions behind the wiring diagrams (e.g. which RFs are most likely to cooperate with each other). In this study, we use data derived from ChIP-Seq and RNA-Seq experiments to predict the cooperative patterns between RFs as they co-regulate the expression of target genes. On a genome-wide scale ChIP-Seq provides regulatory information about wiring between RFs and targets, while RNA-Seq provides gene expression data; by combining these two data types we are able to go beyond the regulatory activities of individual RFs and investigate the relationships between higher order RF groups.

Cells achieve tremendous diversity in their gene expression programs, in large part due to cooperation among RFs, which may individually act as activators or repressors [9]. While the individual activity of many RFs remains to be characterized, their combined actions determine the expression pattern of their target gene. Here, we seek to systematically describe RF cooperation using logic models.

At a high level, the gene regulatory network can be regarded as analogous to an electronic circuit insofar as both gene networks and electronic circuits have inputs and outputs related by certain rules. Therefore, we can build on the vast electronics knowledge base to draw useful insights for understanding and probing biological regulation. For example we can apply regulatory combinatorics, a key design principle in electronics, [10] to the study of gene regulation using logic gate models. A logic gate is a discrete, high-level functional module that describes the relationship between a system’s Boolean input and output elements. By applying logic functions to study TF interactions in *E*. *coli* and *S*. *cerevisiae*, Mangan et al.[11] found that the logic gate is a simple but useful framework for understanding regulatory cooperativity among RFs. While this model is not able to capture the very complex regulatory patterns that may be characterized by continuous models [12,13], it is computationally efficient, and it is comprehensive enough to meaningfully describe a large variety of regulatory networks on a genome-wide scale in multiple organisms. Here, we present a computational method that streamlines the process of inferring logical cooperative relationships among RFs without requiring any prior information regarding their individual activity (as activators or repressors). We successfully apply our algorithm towards developing a comprehensive map of gene regulation.

In numerous cases, gene regulation can be regarded as a logical process, described by a logic gate model, where RF expression levels are the input variables and the target gene expression is the output [3,11,14–22]. For example, DNA sequence motifs have been found to work together following standard combinatorial logic (AND, OR and NOT) to match gene expression patterns [23]. By contrast, TFs can indirectly control gene expression without binding to regulatory sequence elements but rather connecting with other bound TFs through protein-protein interactions [2,24]. As such, in order to describe this process we need a more complex logic pattern. In this respect, we use general logic-circuit models to describe the logic operations for regulatory modules, consisting of multiple RFs and their common target genes.

The three basic logic operators, AND, OR, and NOT, can be combined in a variety of ways to describe all possible logical operations [11]. For any two-input-one-output scenario there are 16 possible logic gates (including all possible logic combinations between positive and negative regulators) (Materials and Methods). These logic gates represent a useful and systematic framework for describing complex interactions between RFs and targets. Previous studies took advantage of binarized regulatory data (provided by perturbation experiments, such as TF knock-outs) and Boolean models in order to capture the logic processes that describe the interactions of TFs [25]. The simple binary operations in the Boolean model are computationally efficient for large-scale datasets. However, previous efforts focused only on a small set of TFs and target genes, missing patterns from genome-wide identification and characterization of logic operations in gene regulation. In addition, numerous other important regulatory factors, such as miRNAs and TFs distally bound to target enhancer regions, have been not been covered in previous regulatory analyses.

By combining the activity of RFs and their respective targets on a genome-wide scale, a bigger picture emerges: the gene regulatory network. To better understand this network we explore the interactions among its various components and features. Mathematically, it can be modeled as a directed network with a hierarchical structure comprising of top, middle, and bottom layers [5,26–28]. Previous studies have shown that the middle levels RFs play important roles in gene regulation. Another feature of gene regulatory networks is the network motif. A common motif is the feed-forward loop (FFL), which consists of two RFs acting on a common target, while one RF regulates the other. FFLs can be classified into eight types based on the combination of the two RFs acting as activators and/or repressors. Previous studies in yeast [11] looked at a small set of FFLs and have shown that they interact following logic operations. Thus, it is interesting to investigate how the logic operations associate with various regulatory network features.

In this paper, we present a novel computational method, Loregic, which integrates gene expression and regulatory data to characterize RFs on a genome-wide scale using logic-circuit models. Loregic classifies individual regulatory factors into functional modules (i.e., regulatory triplets) and reveals how these modules act functionally as logic circuits. We apply Loregic to study regulatory factors (TFs and miRNAs) in the yeast cell cycle and human cancer datasets. We also illustrate our method’s applicability to predict logical cooperation for two regulatory features: indirectly bound TFs and FFLs.

## Results

**Fig 1:**
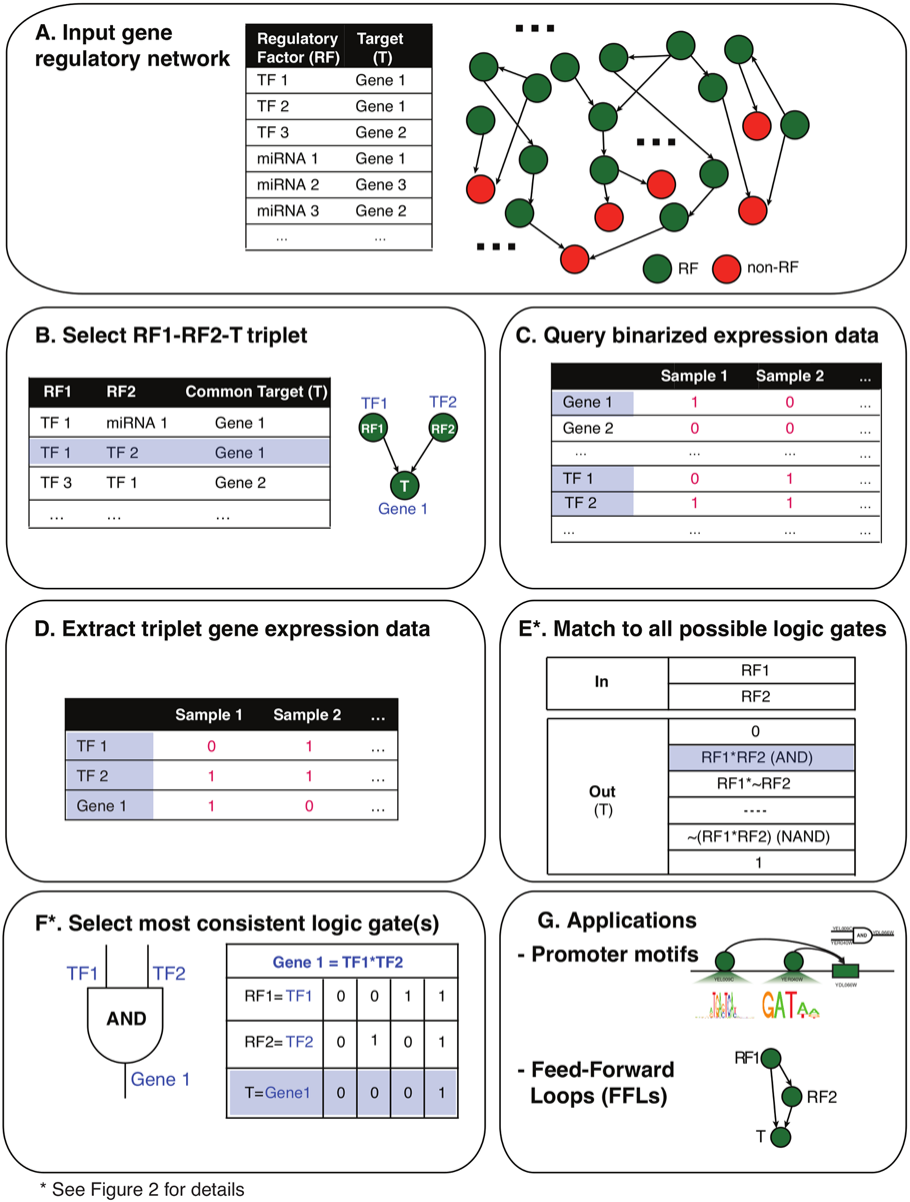
Loregic workflow. **A**: Loregic first inputs a gene regulatory network that consists of regulatory factors and their target genes; **B**: Next, it identifies all possible RF1-RF2-T triplets where RF1 and RF2 co-regulate the target gene T. Note that T can be also a RF; **C**: Loregic queries binarized gene expression data for each triplet, and **D**: it extracts the triplet’s binarized gene expression data; **E**: Loregic matches the triplet’s gene expression against all 16 possible two-input-one-output logic gates based on the binary values, and **F**: finds the matched logic gate if the triplet is gate-consistent, and calculates the consistency score. Then, Loregic repeats steps **C-F** for all triplets from Step **B** in the regulatory network and finds all logic-gate-consistent triplets. In Step **G**, the gate-consistent triplets can be further mapped onto other regulatory features such as: 1) indirectly bound TFs and 2) feed-forward loops.

**Fig 2:**
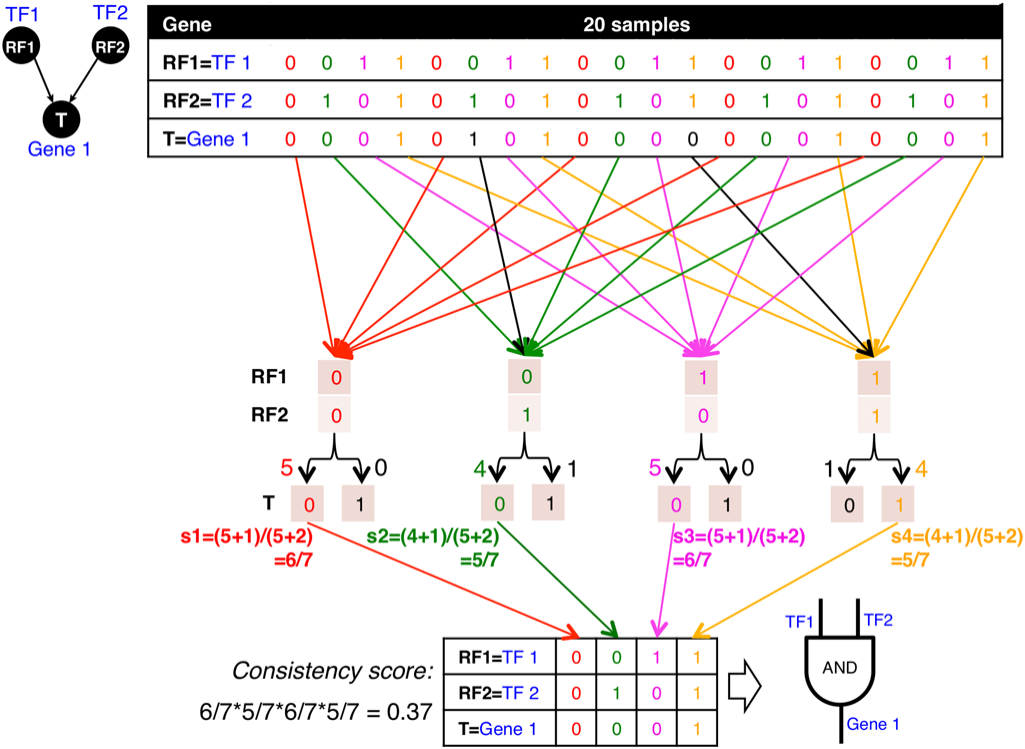
Procedures for mapping logic gates and calculating consistency scores. In this mock example we have binarized expression values for an RF1-RF2-T triplet across a dataset of 20 samples; i.e., *m* = 20 binary vectors. There are 5 vectors with RF1 = 0 and RF2 = 0, all of which have output of T = 0 (red), so (RF1 = 0, RF2 = 0, T = 0) is chosen as the most suitable triplet-logic gate match, and its succession probability *s*_1_ = (5+1)/(5+2) = 6/7 with *n*_1_ = 5 and *m*_1_ = 5 by Laplace’s rule of succession. Next, there are 5 vectors with RF1 = 0 and RF2 = 1, four of which have output of T = 0 (green), and one of which has output of T = 1. We choose (RF1 = 0, RF2 = 1, T = 0) as the most common triplet with its succession probability *s*_2_ = (4+1)/(5+2) = 5/7 with *n*_2_ = 4 and *m*_2_ = 5, because for the given input the majority of cases have zero as the output value. Similarly, when RF1 = 1 and RF2 = 0, T = 0 is chosen (magenta) because it appears more than T = 1, and its succession probability *s*_3_ = (5+1)/(5+2) = 6/7 with *n*_3_ = 5 and *m*_3_ = 5. Finally, when RF1 = 1 and RF2 = 1, T = 1 is chosen (orange) because it appears four times while T = 0 appears only once, and its succession probability *s*_4_ = (4+1)/(5+2) = 5/7 with *n*_4_ = 5 and *m*_4_ = 5. Combining the outputs chosen for four different input combinations of RF1 and RF2, we obtain the triplet’s truth table, and find that it best matches the AND logic gate. As such we consider this triplet to be consistent with the AND gate, and calculate its consistency score to be *C* ^AND^ = *s*_1_ *s_2_ *s_3_ *s_4_ = 0.37.

Loregic takes as inputs two types of data: a regulatory network (defined by RFs and their target genes) and a binarized gene expression dataset across multiple samples. The binarized gene expression data (1-on and 0-off) is simple but useful in representing the network RFs’ activities on target genes. The inputs can be chosen from different resources to meet the user’s needs. In this paper, we used BoolNet [29] to obtain binarized gene expressions. Loregic describes each regulatory module (triplet) using a particular type of logic gate; i.e. the gate that best matches the binarized expression data for that triplet across all samples. Formally, a triplet is described as RF1-RF2-T, where RF1 and RF2 are regulators (e.g. TFs) and T is the target. Note, however, that T itself could be a regulator participating in another triplet. Loregic scores the agreement between the triplet’s cross-sample expression and the idealized expression pattern of each of 16 possible logic gates using Laplace’s rule of succession (Materials and Methods). A high score implies a strong cooperation between the activities of the two RFs on the target as described by the matched logic gate. If such a logic gate is found, we define the triplet as “consistent” with the respective logic gate (i.e. the triplet is described as “logic-gate-consistent” or “gate-consistent”). In the case that no best-matching logic gate is found (e.g. all logic gates score low, or there are tied scores between multiple logic gates), we define the triplet as inconsistent with all logic gates (i.e., “gate-inconsistent”). This negative result suggests that the two-input-one-output model cannot appropriately describe the gene regulation, perhaps due to the fact that more RFs are involved and thus a more complex model should be used (Discussion). In this paper, we evaluate Loregic’s ability to analyze transcription factors, miRNAs and their target genes. In detail, our method comprises of six major steps (Fig. 1):

Step A Input gene regulatory network consisting of regulatory factors and their target genes;
Step B Identify all RF1-RF2-T triplets where RF1 and RF2 co-regulate the target gene T (note that T can also be an RF);
Step C Query binarized gene expression data for each triplet;
Step D Extract the triplet’s gene expression data;
Step E Match the triplet’s gene expression against all possible two-input-one-output logic gates based on the binary values;
Step F Find the matched logic gate if the triplet is gate-consistent, and calculate the consistency score (Fig. 2);
Finally, Steps C-F are repeated for all triplets in the regulatory network, and all logic-gate-consistent triplets are identified.

The gate-consistent triplets can be further mapped onto other regulatory features (Discussion). In this paper we describe two applications leveraging the logic-gate-consistent triplet data: 1) prediction of logic operations for 1) indirectly bound TFs and 2) feed-forward loops.

### Applications

We studied Loregic’s ability to characterize gene regulation in both small and complex biological systems. In particular we analyze two datasets: the cell cycle dataset in yeast, a common model organism, and a well characterized human leukemia dataset.

Yeast (*S*. *Cerevisiae*) constitutes a small but well-studied biological system. The large variety of publicly available gene regulation and expression data makes yeast an ideal model organism to test and validate our algorithm. As an example, we use Loregic to predict logic cooperations among TFs. We validate our results using data from genome-wide TF knockout experiments.

By contrast, human cancers are much more complex biological systems and we use them to illustrate how Loregic can accommodate many different types of regulators (e.g. TFs, miRNAs, distal regulators) within the same framework. Specifically, we use Loregic to study acute myeloid leukemia (AML), a quickly progressing cancer with low five-year survival rates (<25%), expected to cause over 10,000 deaths in the USA in 2014 [30]. We extracted gene regulatory network data from the ENCODE leukemia cell line, K562, and gene and miRNA expression datasets for AML from TCGA.

**Fig 3:**
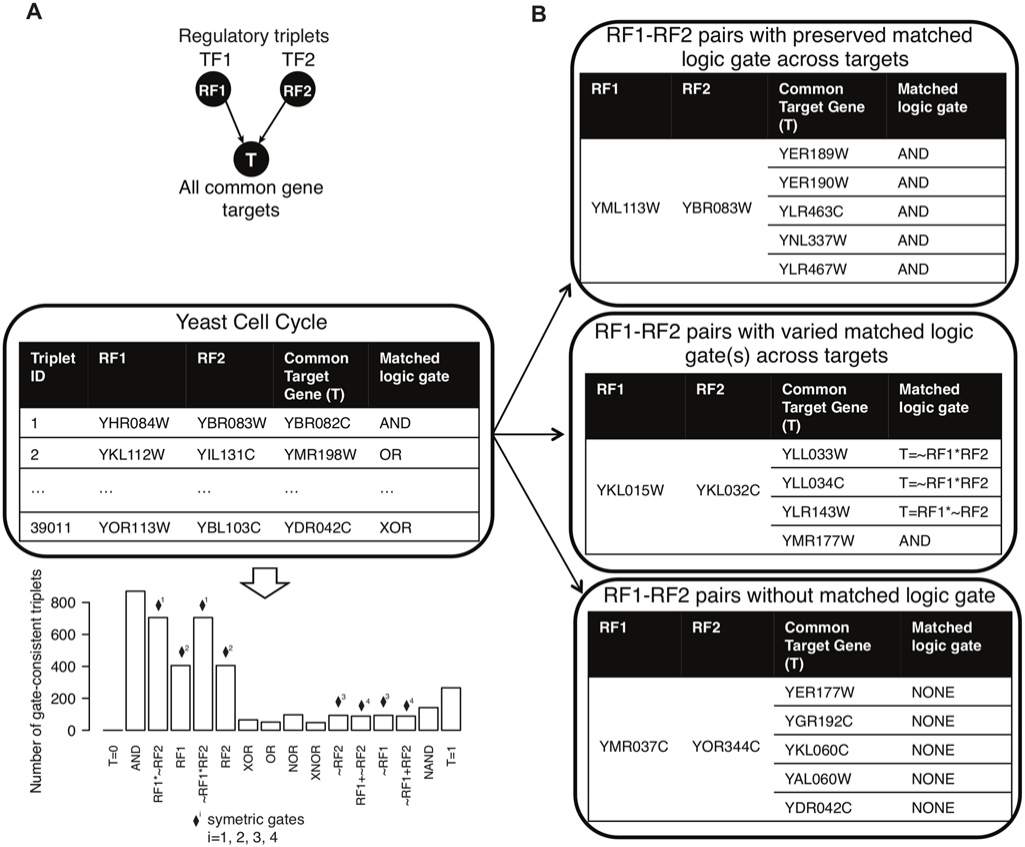
Cooperative logics found by Loregic for yeast regulatory triples. **A**—Loregic gives for each triplet a matched logic gate as shown in the table. The bar plot shows the distribution of 4126 gate-consistent TF-TF-target triplets across matched logic gates. The symmetric gate pairs are marked using diamonds on top of bars with identical superscript numbers due to randomly assigning TFs as TF1 or TF2. **B**—Top: an example RF pair (RF1 is YML113W, RF2 is YBR083W) with “homogenous” gate-consistent triplets—matching the same, logic gate across all targets; middle: an example RF pair (RF1 is YKL015W, RF2 is YKL032C) with “inhomogeneous” gate-consistent triplets—matching different logic gates across all targets; and bottom: an example RF pair (RF1 is YMR037C, RF2 is YOR344C) with non-gate-consistent triplets, i.e. triplets inconsistent with all logic gates across all targets.

**Yeast TFs are cooperative during cell cycle**. We used Loregic to characterize the TF-TF-target logics during the yeast cell cycle (Materials and Methods) and found 4,126 TF-TF-target triplets that are gate-consistent (Fig. 3A, S1 Table). There are totally 39,011 TF-TF-target triplets with 2464 unique targets in yeast cell cycle data. The 4,126 gate-consistent triplets include 757 unique targets. Among the gate-consistent triplets, we found that “T = RF1*RF2” (i.e., AND gate), “T = ~RF1*RF2”, and “T = RF1*~RF2” logic gates, have more triplets matched than all other gates, where ‘~’ and ‘*’ represent the NOT and AND logic operators respectively. It is worth noting that, having randomly assigned TFs as RF1 and RF2, the “T = ~RF1*RF2” and “T = RF1*~RF2” logic gates are symmetric. The AND gate triplets indicate that both TFs are required in order to activate the expression of their target gene (see discussion of other logic gates in S1 Fig). After matching all triplets against logic gates, we looked at variations in matched logic gates for particular types of triplets (RF1, RF2, X), that share regulatory factors (RF1 and RF2) but have distinct targets (T = X) (Fig. 3B). As a result we were able to distinguish three categories for this triplet group: 1) “homogenous” gate-consistent triplets—matching the same logic gate across all targets (e.g., top table); 2) “inhomogeneous” gate-consistent triplets—matching different logic gates across all targets (e.g., middle table); and 3) non-gate-consistent triplets, i.e. triplets inconsistent with all logic gates across all targets (e.g., bottom table).

**Fig 4:**
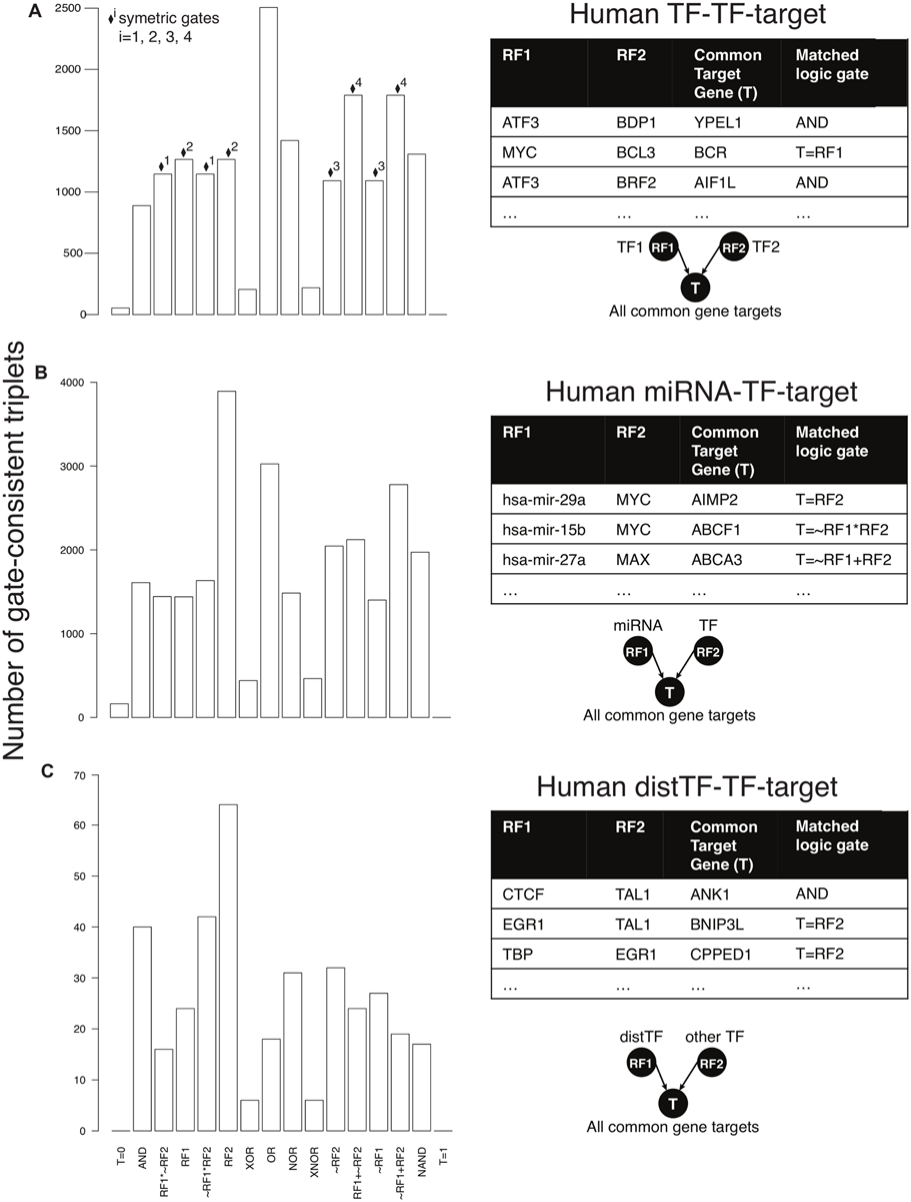
Distributions by logic gate of all gate-consistent human regulatory triples in acute myeloid leukemia. **A—**TF-TF-target triplets. The symmetric gate pairs are marked using diamonds on top of bars with identical superscript numbers; **B**—miRNA-TF-target triplets; **C**—distTF-TF-triplets. The triplets from **B** and **C** have different distributions from **A**, including notably at symmetric gates because their RF1s are miRNA/distTF. Also, the “T = RF2” gate matches more triplets than any other gate in **B** and **C**.

**Logic operations between TF-TF, miRNA-TF, and distTF-TF across targets in acute myeloid leukemia**. Next, we characterized TF-TF, miRNA-TF, and distTF-TF logic operations by integrating ENCODE and TCGA AML datasets using Loregic, where distTF represents a TF regulating its target through a distal regulatory region such as an enhancer, whereas the canonical TF regulation is assumed to occur at the proximal promoter (Materials and Methods, and S2–4 Tables). In total, we identified 50,865 TF1-TF2-target triplets and 821 distTF-TF-target triplets. By integrating miRNA-targets data (Materials and Methods), we were able to identify 56,944 miRNA-TF-target triplets, in which RF1 is an miRNA, RF2 is a TF, and the target is a gene co-regulated by the respective miRNA and TF. Fig. 4 shows the distributions, by logic gate, of these gate-consistent triplets. For example in Fig. 4A, we found that the gate-consistent TF-TF-target triplets preferentially match the OR gate (2505 triplets). The gate-consistent triplets from TF-TF-target, miRNA-TF-target, and distTF-TF-target include 1005 (~55% of 1824 unique targets from 50,865 TF-TF-target triplets), 1672 (~76% of 2210 unique targets from 56,944 miRNA-TF-target triplets), and 66 (~58% of 113 unique targets from 821 distTF-TF-target triplets) unique targets, respectively (Materials and Methods).

In the 16 logic gates, RF1 and RF2 are not necessarily symmetric. To test the relative balance of TFs, miRNAs, and distTFs as regulators in RF1-RF2-target triplet, we randomly assigned TFs as RF1 and RF2, and looked at the variations between potentially symmetrical logic gate pairs (e.g. T = RF1+~RF2 vs T = ~RF1+RF2 or T = RF1 vs T = RF2) in terms of matched triplets. As expected, we found no significant differences for the TF-TF-target triplet (Fig. 4A). However the miRNA-TF-target and distTF-TF-target triplets are different (Fig. 4B and 4C), suggesting that miRNAs and distTFs (as RF1) interact with TFs (as RF2) following different regulatory logics. For these scenarios, the “T = RF2” gate matches more triplets than any other gate, suggesting that in AML the dominant regulators of target expression are the promoter-binding TFs rather than miRNAs or distTFs.

### Validation

We assessed the biological relevance of the insights into gene regulation gained by using logic circuit models, by comparing our results with experimental observations described in literature for yeast and human regulatory factors.

**Deleting TFs that form cooperative logic gates gives rise to significantly higher fold changes of target gene expression**. We used yeast genome-wide TF knockout experiments to validate the TF logic from gate-consistent triplets. The yeast TF knockout experiments give information regarding fold changes in gene expression as a result of deleting a single TF [31,32]. Using these knockout datasets, we found that if a target gene is regulated by two cooperative TFs in an AND relationship, and thus it is most likely that the presence of both TFs is required to turn on the target gene (S1 Fig), the deletion of either TF impacts the target expression. For example, in analyzing 871 AND-gate-consistent triplets, we found that deleting either of their TFs gave rise to substantial down-regulation of their target genes, i.e., the logarithm expression fold changes were significantly less than zero (*t-test p-value* = 0.068). For the triplets consistent with non-cooperative gates such as “T = RF1” or “T = RF2” (i.e., only one TF controls the target regulation), we found that the target gene is more affected (down-regulated) by the removal of the dominant RF (i.e., RF1 for “T = RF1” consistent triplets, RF2 for “T = RF2” consistent triplets) than the removal of the other one (*t-test p-value* < 0.0004 for 811 triplets consistent with “T = RF1” or “T = RF2”).

**Fig 5:**
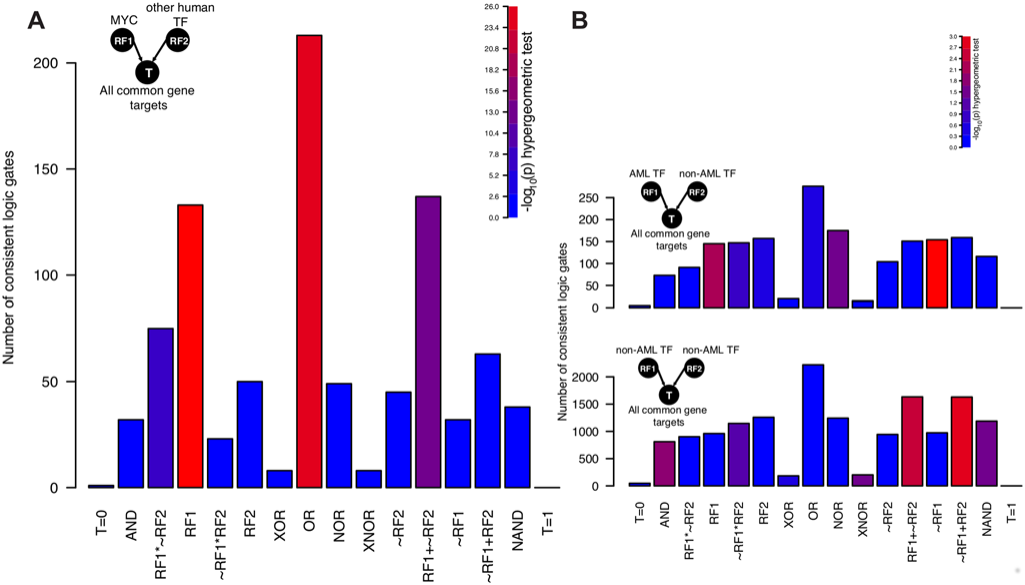
Distributions by logic gate of gate-consistent human regulatory triples associated with AML-related TFs. The bar color represents-log_10_(hyper-geometric enrichment p-value) (Materials and Methods). **A—**The triplets in which RF1 is MYC, RF2 is chosen from other human TFs, and T is a common target. The two most enriched logic gates are “T = RF1” (133 triplets, hyper-geometric test *p*(133, 2153, 1110, 50865)< 4.3*10^-27^) and “T = RF1+RF2 (OR)” (211 triplets, hyper-geometric test *p*(211, 2153, 2505, 50865)< 1.1*10^-21^), which supports the finding that MYC is a universally amplifier for its target expression; **B—**the triplets in which RF1 is chosen from AML-related TFs, RF2 is chosen from TFs not relating to AML, and T is a common target as shown in top, and the triplets in which both RF1 and RF2 are chosen from TFs not relating to AML, and T is a common target as shown in bottom. “T = RF1” and “T = ~RF1” are the two most enriched matched logic gates when RF1 is AML-related TF, which implies that AML-related TFs dominate the regulation of their target expression.

**AML-related TFs play a dominant role in regulating target gene expression**. Next, we showed that Loregic can make interpretable gate assignments for a cancer-related TF, MYC, which has been found to universally amplify target gene expressions in lymphocytes [33]. We identified 2,153 MYC-TF-target triplets (i.e., RF1 is MYC, RF2 is chosen from other TFs from ENCODE, and T is target), and found that 905 of them are gate-consistent. The two most enriched logic gates are “T = RF1” (133 triplets, hypergeometric test p-value < 4.3*10^-27^) and “T = RF1+RF2 (OR)” (211 triplets, hypergeometric test p-value < 1.1*10^-21^) (Fig. 5A). For the 133 triplets consistent with “T = RF1” with RF1 being MYC, our model predicted that high expression of MYC is necessary and sufficient for high target gene expression. For the 211 triplets consistent with “T = RF1+RF2” with RF1 being MYC and RF2 being other TFs, our model predicted that high expression of MYC is sufficient but not necessary for high target expression. Both of the most commonly observed scenarios indicate that high MYC expression is sufficient for high target expression. These results support the recent findings that MYC plays a universal amplifier role in gene expression.

Finally, we analyzed all the triplets associated with all AML-related TFs, where RF1 is chosen from AML-related TFs, RF2 is chosen from non-AML related TFs, and T is their common target. The AML-related TFs were TFs whose genes have been observed as mutated in some AML sample [34]. We found that “T = RF1” and “T = ~RF1” (Fig. 5B) are the most enriched matched logic gates for these TFs. However, we did not find any enrichment for these two gates in triplets containing only non-AML TFs. These results suggest that the AML-related TFs play a dominant role in regulating target expression in this cancer.

### Loregic applications for other regulatory features

**Fig 6:**
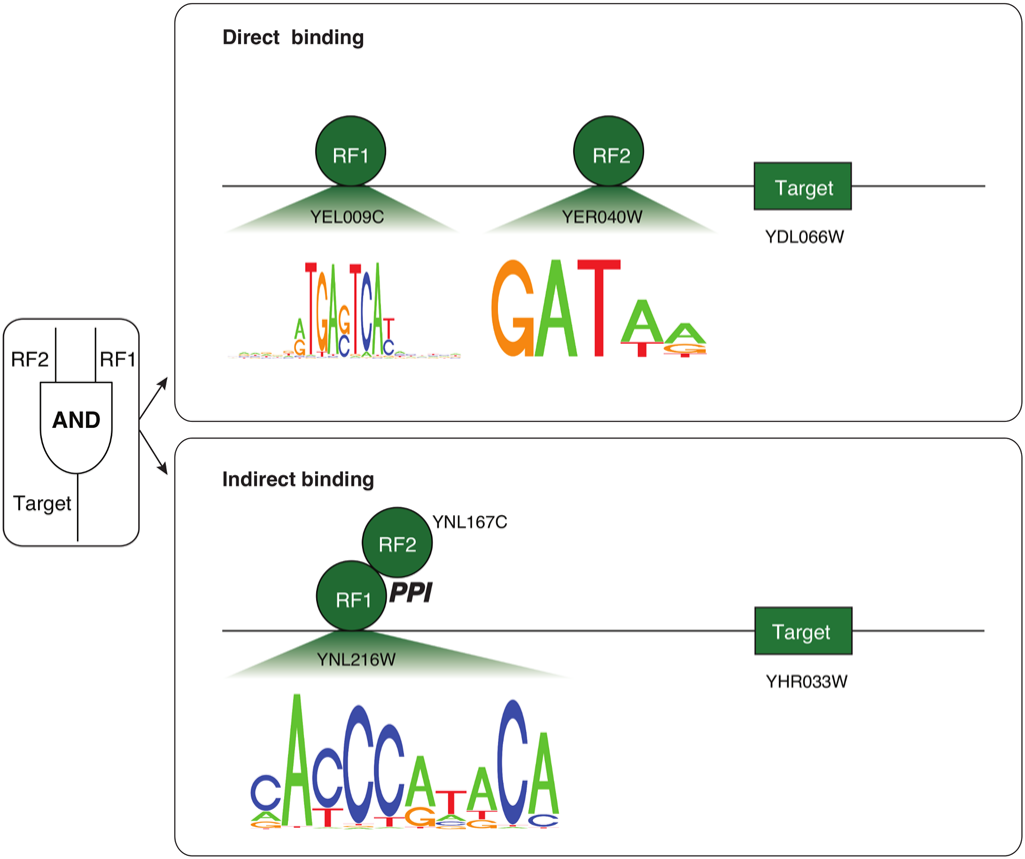
Promoter motifs for AND-consistent yeast triplets with directly and indirectly bound TFs. We present two example yeast triplets, (RF1 is the TF YEL009C, RF2 is the TF YER040W, T is the gene YDL066W) at top and (RF1 is the TF YNL216W, RF2 is the TF YNL167C, T is the gene YHR033W) at bottom, both of which are consistent with the AND gate by Loregic. Both TFs in the top triplet have motifs in the target promoter region, but only one TF, YNL216W, in the bottom triplet has a motif in the target promoter region. The other TF, YNL167C, cooperates with YNL216W in an AND logical relation via protein-protein interaction.

**Classification of logic-gate-consistent triplets with indirectly bound TFs**. TFs can regulate target genes without binding directly to regulatory regions by instead forming protein-protein interactions with already bound TFs [2]. We suggest that evaluating the cooperative logic of TF pairs along with the analysis of promoter motifs can give insights regarding this type of TF-binding activity. We studied TF promoter motifs in target promoter regions (1,000 bps in yeast and 5,000 bps in human upstream of the transcription start site) [35–38]. In a number of cases even when the logic gate assessment predicted cooperation between the two TFs, we could not find a binding motif for one of these TFs (i.e., a Position Weight Matrix) in the target gene’s promoter region. This suggests that the motif-missing TF is only involved with the target gene indirectly—perhaps through a protein-protein interaction (specifically for this assessment, we define a TF binding motif are missing if we couldn’t find any matches in target promoter sequences for TF motifs with at least 80% Position Weight Matrix (PWM) similarity using matchPWM(…, min.score = “80%”) in [39]. Out of 948 yeast TF-TF-target triplets consistent with “T = RF1*RF2” (AND gate) (Fig. 6), 348 have one TF whose motif is not present in the target’s promoter region. The same holds true for 364 out of 1,100 for “T = RF1*~RF2” and 377 out of 1,095 for “T = ~RF1*RF2”, a symmetric logic gate pair. Similarly, in the human leukemia dataset, we found that from 888 TF-TF-target triplets consistent with AND gates, 71 have one TF whose motif is not present in the target’s promoter. For example (S2 Fig), the triplet of (RF1 is USF2, RF2 is NFYB, T is YPEL1) is consistent with the AND gate, and both TFs have motifs in the YPEL1 promoter region. By contrast, the AND-consistent triplet, (RF1 is USF2, RF2 is NFE2, T is NBPF1) has a USF2 motif but not an NFE2 motif in NBPF1’s promoter region, which is explained by reports that USF2 and NFE2 are connected through protein-protein interactions and that NFE2 regulates NBPF1 through indirect binding [2]. As such, it is possible that those TFs with absent motifs (as above) can potentially regulate their targets by cooperating with directly bound TFs through protein-protein interactions, a phenomenon that has been previously observed [2,24,40–42]. Moreover, we further classified those triplets with indirectly bound TFs using their matched logic gates, and identified the indirectly bound TFs cooperating with bound TFs to regulate their targets in a logical way (S5 and S6 Tables).

**Logic gates for feed-forward loops**. Feed-forward loops (FFLs) are RF1-RF2-T triplets in which RF1 also regulates RF2. FFLs have been found to be important motifs in regulatory networks, with many interacting by following logic operations [11]. We apply Loregic to find the logic operations that characterize the FFLs from a genome-wide perspective in both the yeast cell cycle and human leukemia cancer datasets. For the yeast regulatory network, we found that from a total 5707 FFLs, 659 constitute gate-consistent triplets. Out of these, 162 are consistent with the AND gate (with enrichment by hypergeometric test p-value <1.3*10^-3^), and 159 are consistent with “T = RF1” (with enrichment by hypergeometric test p-value <7.5*10^-5^) making them the dominant logic gates for yeast FFL. These results match previous experiments that have shown that the majority of FFLs are of the so-called coherent type 1, in which RF1 activates RF2, and both activate the target [11].

Next, we looked at FFLs from human leukemia TF-TF-T triplets (23,385 FFLs in total), and found that the two most abundant matched logic gates are “T = RF1” (1,306 FFLs with enrichment by hypergeometric test <3.4*10^-9^) and “T = RF1+~RF2” (1,765 FFLs with enrichment by hypergeometric test <1.7*10^-5^). Both gates match the logics of the coherent type 4 FFL, where RF1 down-regulates RF2, RF2 down-regulates target, and RF1 activates target as described in [11]. This suggests that the master TF (RF1) of the FFL aims to activate the target, but due to the gene down-regulation action from the secondary TF (RF2), it must simultaneously down-regulate RF2. Moreover, we did not find any enriched logic gates among the triplets that do not form FFLs in both yeast and human.

**miRNAs and MYC down-regulate each other**. MYC (described above) and miRNAs have been found to down-regulate each other by forming double down-regulatory FFLs in leukemia [43]. We identified 1,805 miRNA-MYC-target triplets with 117 miRNAs, 1,143 of which are gate-consistent. From these triplets, 446 match “T = RF2” when RF2 is MYC (hypergeometric test p-value < 2.5*10^-124^), and 201 match “T = ~RF1+RF2” when RF1 is a miRNA and RF2 is MYC (hypergeometric test p-value < 4.1*10^-25^). These two dominant logic gates also match the logic for the coherent type 4 FFL as described in [11]. As expected, these results imply that miRNAs repress target gene expressions, while MYC activates target gene expressions and simultaneously down-regulates the miRNAs. We also found 56 gate-consistent miRNA-MYC-target triplets matching “T = ~RF1*RF2” when RF1 is a miRNA and RF2 is MYC, and 16 triplets matching “T = ~RF1” with RF1 being a miRNA. These two logics match the coherent type 2 FFL[11]. This result suggests that miRNAs repress the expression of both MYC and the target gene, while MYC activates the target. In short, these matched logic gates support the notion that the miRNAs and MYC form a double-negative regulatory loop in this system.

**Fig 7:**
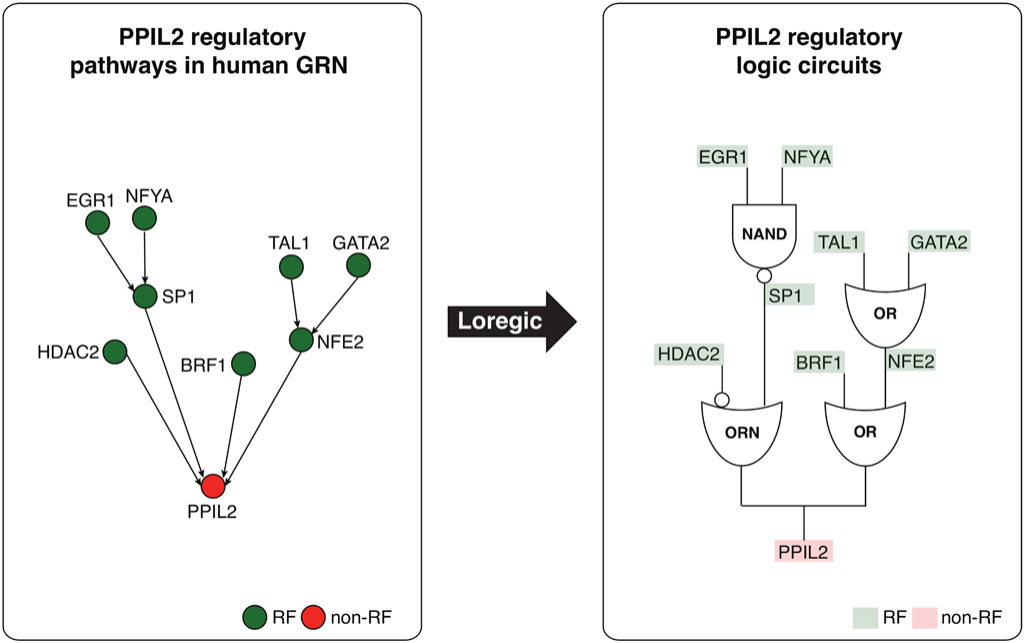
Depiction of two logic circuit regulatory pathways targeting PPIL2. Two logic circuit regulatory pathways targeting the PPIL2 gene, an important cyclophilin member in immunological suppression, are found by Loregic: **1**: PPIL2 is co-regulated by HDAC2 and SP1 forming the triplet of (RF1 is HDAC2, RF2 is SP1, T is PPIL2), which is consistent with the “T = ~RF1+RF2” gate (the ORN gate[22]), and SP1 is co-regulated by EGR1 and NFYA forming the triplet of (RF1 is EGR1, RF2 is NFYA, T is SP1), which is consistent with the “T = ~RF1*~RF2 (the NAND gate); **2**: PPIL2 is also co-regulated by BRF1 and NFE2 forming the triplet of (RF1 is BRF1, RF2 is NFE2, T is PPIL2), which is consistent with OR gate, and NFE2 is co-regulated by TAL1 and GATA2 forming the triplet of (RF1 is TAL1, RF2 is GATA2, T is NFE2), which is also consistent with OR gate. We replace the triplets on these pathways using matched logic gates, and depict the pathways using logic circuits to summarize the regulatory logics targeting PPIL2 at the pathway level.

**Logic circuit analysis of regulatory pathways and hierarchies**. Target gene expression is a complex process controlled by multiple RFs whose own expressions are in turn dictated by other RFs, forming regulatory pathway cascades. Analyzing these pathways in a systematic fashion, we are able to get a comprehensive picture of a particular gene regulation. Going beyond the prediction of the cooperative logics of individual RFs that directly regulate a target, we are interested in identifying all the logic gates matching triplets involved in the target regulatory pathway, in order to obtain a coherent logic circuit pathway. This logic circuit pathway depicts the logical relationship between all RFs in the regulatory pathway of a target gene. For example in Fig. 7A, Loregic found that there are two regulatory pathways regulating the target gene, PPIL2 in human K562. PPIL2 is an important cyclophilin member in immunological suppression These two pathways contain 4 logic-gate-consistent triplets forming a two-layer hierarchical structure. By replacing these triplets with corresponding matched logic gates, we obtain the logic circuit regulatory pathways for the target gene (Fig. 7B). Studying the circuit logic gates, we are able to deduce the Boolean logic equation that describes the logical relationship between all RFs in the regulatory pathways of the target gene. In addition, a variety of regulatory pathways can be connected to form gene regulatory networks, which have the hierarchical structures [5,26–28]. TFs typically lie at higher layers than non-TFs in hierarchical gene regulatory networks. We found that the consistency scores of the triplets with TF targets are significantly greater than the ones with non-TF targets in both yeast and human (K-S test p value < 4e-6 in yeast and < 0.04 in human), which implies that the regulatory co-operation at higher hierarchical layers more likely follows a clear “logical pattern”. We also constructed the regulatory hierarchical networks with top, middle and bottom layers using yeast TFs [5], and found that the scores for the gate-consistent triplets targeting the bottom TFs are lower than the ones targeting middle and top TFs (S3 Fig), which implies that the regulations of middle and top TFs more likely follow logical operations than the bottom TFs.

## Discussion

Loregic is a multi-purpose computational method that uses logic-circuit models to characterize the cooperativity among regulatory factors such as TFs and miRNAs by integrating gene expression and regulatory network data. Given the multitude of high quality expression (e.g., RNA-seq, small RNA-seq), and regulation (e.g., ChIP-seq, CLIP-seq, DNase-seq) datasets available, Loregic can be further used to study cooperations among other regulatory elements such as splicing factors, long non-coding RNAs, etc., or RF cooperations during other biological processes such as embryonic developments for the model organisms in modENCODE project [44]. To our knowledge, the present study describes for the first time the use of 16 logic operations to perform a comprehensive genome-wide analysis of regulatory triplets.

We tested Loregic using two-RFs-one-target triplets, focusing on scenarios where the RFs are either two TFs or one TF and one miRNA or distTF. However, we can extend Loregic to analyze regulatory modules with multiple RFs and multiple target genes using higher-order logic circuit models discussed as above if there is enough supporting data. Loregic is also compatible with other discretization methods including using any custom-made binarized gene expression data as input. Loregic uses the gene expression dynamics across samples at the logical level to characterize the regulatory cooperativity. To capture the logical cross-sample dynamics, we recommend the binarization methods such as BoolNet [29]. Unlike traditional ways using a uniform cutoff strategy such as median value, this method customizes the binarization cutoff for each gene based on its expression dynamic patterns across samples. We compared BoolNet with another method, ArrayBin [45], which uses an adaptive approach to binarize high-throughput gene expression data (Materials and Methods).

In our analysis, we found triplets that were inconsistent with all the logic gates. There are several potential explanations for such cases. First, the cooperative patterns of two RFs might follow a more complex mechanism, perhaps one that depends on timing or the phosphorylation state of the RF, which our model does not take into account. Second, the target gene might be regulated by more than two RFs, and thus a higher-order logic circuit model with multiple inputs (>2) as discussed above might be required to capture the RF-target logic. Finally, the target gene expression may also be impacted by stochastic signals, which may necessitate more advanced models such as Fuzzy logic models [12,46].

One of Loregic’s functionalities is relating triplet logics to any set of regulatory network features. Here, we map the logic-gate-consistent triplets to two regulatory features: promoter sequence motifs and feed-forward loops. Loregic’s results can also be directly applied to differentially assess the abundance of various types of logic gates among other gene regulatory features such as regulatory hierarchies we analyzed in this paper. A potential future application would be identifying logic cooperations between and among RFs at different hierarchical layers in the network, which may potentially help understand cooperations among even larger order regulatory groups. In addition, we can use a series of cascaded logic-gate-consistent triplets to capture the logical cooperations of more complex hierarchical structures such as regulatory pathways across multiple layers in hierarchical gene regulatory networks.

In summary, Loregic systematically characterizes genetic regulatory cooperativity using logic-circuit models. This algorithm is widely applicable for the study of regulatory mechanisms and to the assembly of the full panoramagram of gene regulatory activity.

## Materials and Methods

### Gene expression, transcription factor and miRNA datasets

We analyzed the gene expression in yeast using three well-studied cell-cycle datasets: 1) alpha-factor time course with 18 time points (0, 7’, …, 119’); 2) cdc15 time course with 24 time points (10’, 30’, …, 290’) and 3) cdc28 time course with 17 time points (0, 10’, …, 160’) [47,48]. We combined all three datasets (5,581 genes and 59 time points) and normalized gene expressions for each time point by centering the mean to zero. For gene regulation in yeast, we used 176 transcription factors with their target genes identified in [49,50], and found 39,011 TF-TF-target triplets with 2464 unique targets.

In the study of gene expression in human leukemia, we obtained RNA-seq RPKM expressions from The Cancer Genome Atlas Data Portal [51] for 19,798 protein-coding genes and 705 miRNAs across 197 and 188 AML samples, respectively. For each sample, we standardized the log(RPKM+1) across all genes. We identified 50,865 TF1-TF2-target triplets with 1824 unique targets using ChIP-seq data (70 TFs) from ENCODE K562 cell line [5] and 821 distTF-TF-target triplets with 113 unique targets, where distTFs were predicted to bind distal regulatory regions [52]. By integrating miRNA- and TF-target pairs in K562, we were able to identify 56,944 miRNA-TF-target triplets with 2210 unique targets in which RF1 is an miRNA, RF2 is a TF, and the target is their co-regulated gene. The miRNA-target pairs that we used for human K562 cell line in this paper were the overlapped pairs among widely used public databases for predicting miRNA-target relationships described in [53].

### Converting gene expression changes over conditions to Boolean values

In this paper, we binarized the gene expression levels to Boolean values 1 and 0 to represent high and low gene expression, respectively, using BoolNet. Loregic is also compatible with user-inputted, customized binary gene expression data [29]. BoolNet assigns Boolean values to expression data on the basis of modular co-expression patterns by *k*-means clustering across inputted samples and therefore accounts for differences in the dynamic ranges of expression among genes in the input data. After conversion, we found that in yeast 79% of values are 0 (low expression level) and 21% are 1 (high expression level) while in human 42% of values are 0 and 58% are 1. To test the robustness of Loregic to different binarization methods, we compared BoolNet with another method, ArrayBin [45], which uses an adaptive approach to binarize high-throughput gene expression data. We found that the 81% yeast and 85% human TF-TF-target triplets have the same best-matched logic gates found by Loregic between two binarization methods. Also, the consistency scores for those triplets between two methods are highly correlated (Yeast, cor = 0.80; Human, cor = 0.97) (S4 Fig). Human has higher correlation because it has more data samples (200 samples) than yeast (59 samples).

### Mapping and scoring RF1-RF2-T triplets to 16 logic gates

Mathematically, a logic gate can be described by the truth table that lists the outputs of the logic gate for each allowed combination of inputs. For a two-input-one-output logic gate, each of the two input variables may take on either of the two possible values, 0 or 1; thus, the truth table will contain four binary two-element vectors representing all the possible combinations of the two input variables i.e., *v*_1_ = (0,0), *v*_2_ = (0,1), *v*_3_ = (1,0), *v*_4_ = (1,1), where *v*_*i*_ is the vector representing *i* ^th^ input combination *i* = 1,2,3,4. The truth table output will be a vector with four elements, with each element having two possible values 0 or 1. Thus there are 2^4^ possible combinations of 0 and 1 for the output vector, and hence, there are 16 possible two-input-one-output truth tables. The 16 different truth tables correspond to 16 logic gates as shown in S1 Fig. The three basic logic operations, AND (“*”), NOT (“~”) and OR (“+”) are used to express all the 16 possible logic gates.

We denote *f* ^*g*^(*v*_*i*_) the function to obtain the output value from the *i* ^th^ input vector *v*_*i*_ in the logic gate *g*, with *i* = 1,2,3,4. For example for the AND logic gate we have:

$$f^{\text{AND}}\left(v_{1}\right)=f^{\text{AND}}\left(0,0\right)=0*0=0$$

$$f^{\text{AND}}\left(v_{2}\right)=f^{\text{AND}}\left(0,1\right)=0*1=0$$

$$f^{\text{AND}}\left(v_{3}\right)=f^{\text{AND}}\left(1,0\right)=1*0=0$$

$$f^{\text{AND}}\left(v_{4}\right)=f^{\text{AND}}\left(1,1\right)=1*1=1$$

In our model, the two RFs (RF1, RF2) in a regulatory triplet RF1-RF2-T, serve as inputs, while the common target gene T is the output—the result of the *f* ^*g*^ acting on the (RF1, RF2) binary vector.

For *m* samples, we denote $\vec{x}$, $\vec{y}$, and $\vec{z}$ as the *m*-dimensional binary vectors containing *m* binarized expression values for RF1, RF2, and T respectively. Loregic identifies the logic gate whose truth table best matches the input/output data as follows. For the input vector *v*_*i*_, we denote as $m_{i}=\overset{m}{\underset{j=1}{\sum }}I\left(x\left[j\right]=v_{i}\left[1\right]\right)I\left(y\left[j\right]=v_{i}\left[2\right]\right)$ the number of samples (*x*[*j*], *y*[*j*]) matching *v*_*i*_, where **I**(.) is indicator function, *x*[*j*] and *y*[*j*] are *j* ^th^ elements of $\vec{x}$ and $\vec{y},$ with *j* = 1,2,…,*m*, and *i* = 1,2,3,4. Thus we have *m* = *m*_1_+*m*_2_+*m*_3_+*m*_4_. Second, given a logic gate *g*, we denote

$$n_{i}=\overset{m}{\underset{j=1}{\sum }}I(z\left[j\right]=f^{g}\left(x\left[j\right],y\left[j\right]\right)I\left(x\left[j\right]=v_{i}\left[1\right]\right)I\left(y\left[j\right]=v_{i}\left[2\right]\right)$$

as the number of *z*[*j*], *j* = 1,2,…,*m* target binary samples matching the logic gate *g* output *f* ^*g*^(*v*_*i*_), for the *v*_*i*_ input vector. Next we calculate $s_{i}^{g}$ = (1+*n*_i_)/(2+*m*_i_) as the succession probability matching the *v*_i_ of *g* by Laplace’s rule of succession [54]. The succession probabilities are used to simply but rigorously penalize logic-gate assignments that were distinguished from alternative logic gates on the basis of only a small number of observations. As such, given the binarized expression data,$\vec{x}$, $\vec{y}$, and $\vec{z}$ for the RF1-RF2-T triplet, the consistency score for the logic gate *g*, $C^{g}(\vec{x},\;\vec{y},\;\vec{z})$ is given by the product of the succession probabilities for four input types, $s_{1}^{g},s_{2}^{g},s_{3}^{g},s_{4}^{g}$as follows:

$$C^{g}\left(\vec{x},\vec{y},\vec{z}\right)=\overset{4}{\underset{i=1}{\prod }}s_{i}^{g}\left(\vec{x},\vec{y},\vec{z}\right)=\overset{4}{\underset{i=1}{\prod }}\frac{1+n_{i}}{2+m_{i}}$$

Finally, we choose the logic gate with the highest consistency score as the best matched logic gate for the analyzed triplet. In the event of a tie among two or more logic gates for the highest consistency score, we consider the triplet to be gate-inconsistent. Note that according to Laplace’s rule of succession, if there is no data available for a triplet, then *m* = *n*_*i*_ = *m*_*i*_ = 0, and for each logic gate the consistency score by the succession rule is 1/2*1/2*1/2*1/2 = 1/16, which is the probability of a random guess from 16 logic gates. Fig. 2 exemplifies the calculation of the consistency score for a mock TF1-TF2-T triplet across a dataset of 20 samples. In order to identify and remove potentially spurious logic gate assignments, we calculate a permutation score for each triplet over the 16 logic gates as follows: We suppose that the triplet matches the *k* ^th^ logic gate, *g*_*k*_. We replace the target gene, T by a randomly selected gene *M* times (here we use *M* = 1000), and define its permutation score, as *p*(*g*_*k*_) = (the number of replacement triplets that can be identified as gate-consistent with matched *g*_*k*_)/*M*. A high permutation score implies that random effects may cause the matched logic gate. In this paper, we only keep the gate-consistent triplets with permutation scores less than 0.1.

### Enrichment of particular logic gates among consistent triplets by hyper-geometric test

Given a set of triplets (e.g., the triplets in which RF1 is MYC) and a particular logic gate *g*, we calculate a hyper-geometric enrichment p-value to describe the enrichment of triplets consistent with the gate *g* as opposed to other gates as follows: The p-value is equal to$p\left(k_{g},k,K_{g},N\right)=\overset{k}{\underset{i=k_{g}+1}{\sum }}\frac{\left(\begin{array}{c} K_{g}\\ i \end{array}\right)\left(\begin{array}{c} N-K_{g}\\ k-i \end{array}\right)}{\left(\begin{array}{c} N\\ k \end{array}\right)}$, where *k* is the number of triplets in the set, *k_g_* is the number of triplets consistent with the gate *g* in the set, *K_g_* is the total number of triplets consistent with the gate *g*, and *N* is the total number of triplets.

## Supporting Information

S1 Fig Truth tables of all 16 two-input-one-output logic gates. Each of the four two-row columns of 0s and 1s of the Input block represents one of the four possible combinations of input values to a two-input logic gate. Each of the four columns of 0s and 1s in the Output block reports the output value of each logic gate for the input combination specified in the corresponding column from the Input block. The last column of the Output block summarizes the function of each logic gate in the context of gene regulation.
(TIF)

S2 Fig Promoter motifs for two AND-consistent human triplets in Integrative Genomics Viewer (IGV). We present two example human triplets, (RF1 is USF2, RF2 is NFYB, T is YPEL1) at top and (RF1 is USF2, RF2 is NFE2, T is NBPF1) at bottom, both of which are consistent with AND gate by Loregic. Two TFs in the top triplet have motifs at target promoter region (red and purple bars in IGV), but only one TF, USF2 in the bottom triplet has motif at target promoter region (red bars only in IGV). The other TF, NFE2 cooperates with USF2 in an AND logical relation via protein-protein interaction.
(TIF)

S3 Fig Scores of logic-gate-consistent triplets targeting the transcription factors at top, middle and bottom hierarchical layers in yeast. Boxplot displays the score distributions of the logic-gate-consistent triplets with targets being TFs at three different hierarchical layers: top, middle and bottom. The TFs at bottom have lower scores than others in yeast.
(TIF)

S4 Fig Consistency scores of logic-gate-consistent triplets using the binarized datasets from two methods, BoolNet and ArrayBin. Scatterplots (left: yeast, right: human) display the consistency scores of logic-gate-consistent triplets that best match the same logic gates by Loregic using two binarized datasets: one is from the BoolNet method in this paper (x-axis), and another is from the ArrayBin method (y-axis) [45]. The scores are highly correlated between two methods (correlation = 0.80 in Yeast, and 0.97 in Human).
(TIF)

S1 Table The logic-gate-consistent yeast regulatory triplets of (TF1, TF2, target): Column 1, TF1; Column 2, TF2; Column 3, common target; Column 4, matched logic gate; Column 5, triplet’s con-sistency score to matched logic gate. (TXT)

S2 Table The logic-gate-consistent human regulatory triplets of (TF1, TF2, target): Column 1, TF1; Column 2, TF2; Column 3, common target; Column 4, matched logic gate; Column 5, triplet’s consistency score to matched logic gate. (TXT)

S3 Table The logic-gate-consistent human regulatory triplets of (miRNA, TF, target): Column 1, miRNA; Column 2, TF; Column 3, common target; Column 4, matched logic gate; Column 5, triplet’s consistency score to matched logic gate. (TXT)

S4 Table The logic-gate-consistent human regulatory triplets of (distTF, TF, target): Column 1, distTF; Column 2, TF; Column 3, common target; Column 4, matched logic gate; Column 5, triplet’s consistency score to matched logic gate. (TXT)

S5 Table The cooperative logic-gate-consistent yeast regulatory triplets with indirect binding TFs: Column 1, TF1; Column 2, TF2; Column 3, common target; Column 4, number of TF1 binding motif found at target’s promoter (i.e., 1000 bps upstream of target transcription start site); Column 5, number of TF2 binding motif found at target’s promoter. (TXT)

S6 Table The cooperative logic-gate-consistent human regulatory triplets with indirect binding TFs: Column 1, TF1; Column 2, TF2; Column 3, common target; Column 4, number of TF1 binding motif found at target’s promoter (i.e., 5000 bps upstream of target transcription start site); Column 5, number of TF2 binding motif found at target’s promoter. (TXT)
