# Supplementary figures and images for: Loregic: A Method to Characterize the Cooperative Logic of Regulatory Factors
Source: PLoS Comput Biol. 2015 Apr 17;11(4):e1004132. doi: 10.1371/journal.pcbi.1004132 (PMC4401777; doi:10.1371/journal.pcbi.1004132)

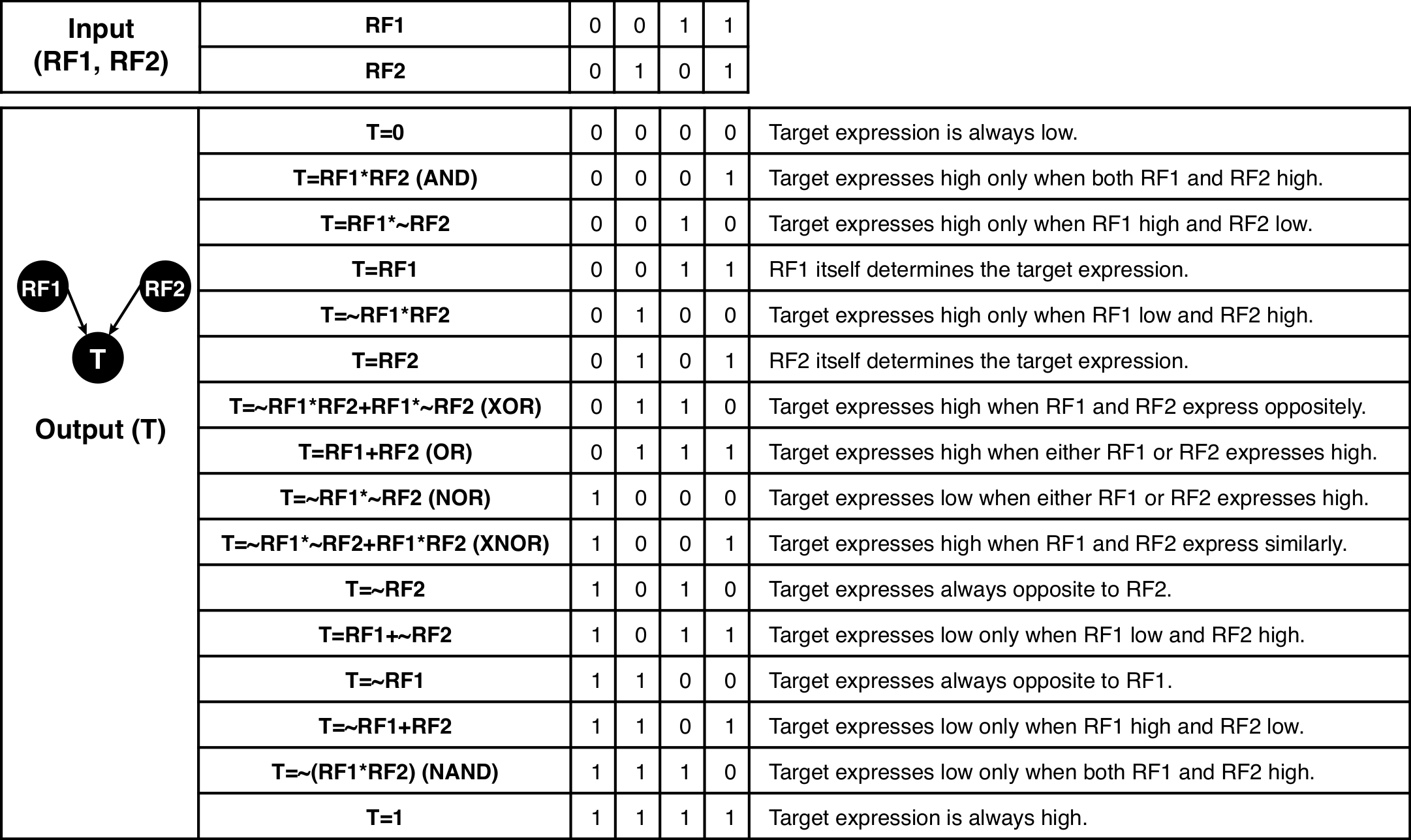

Supplement: S1 Fig — Each of the four two-row columns of 0s and 1s of the Input block represents one of the four possible combinations of input values to a two-input logic gate. Each of the four columns of 0s and 1s in the Output block reports the output value of each logic gate for the input combination specified in the corresponding column from the Input block. The last column of the Output block summarizes the function of each logic gate in the context of gene regulation. (TIF) [file pcbi.1004132.s001.tif]

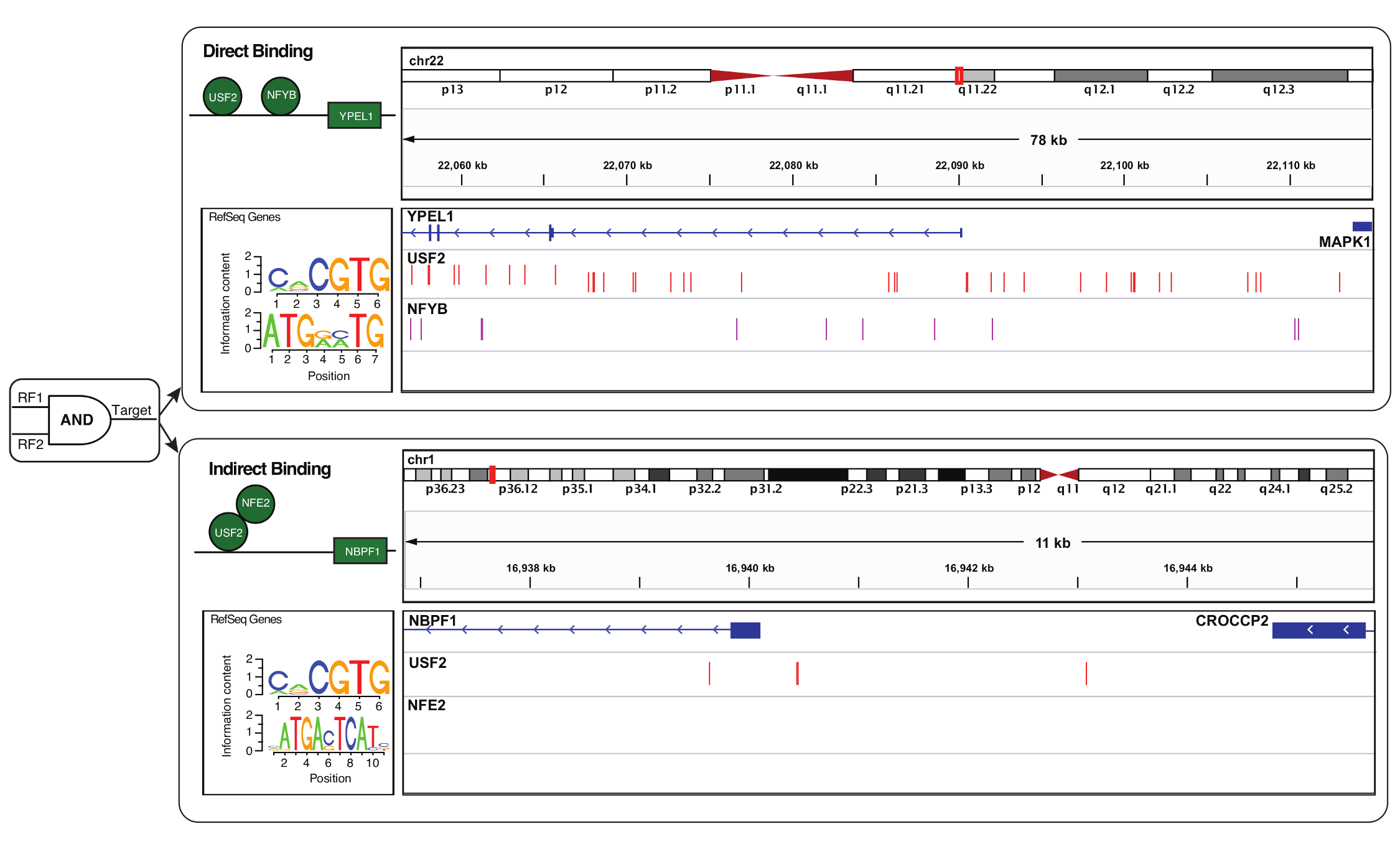

Supplement: S2 Fig — We present two example human triplets, (RF1 is USF2, RF2 is NFYB, T is YPEL1) at top and (RF1 is USF2, RF2 is NFE2, T is NBPF1) at bottom, both of which are consistent with AND gate by Loregic. Two TFs in the top triplet have motifs at target promoter region (red and purple bars in IGV), but only one TF, USF2 in the bottom triplet has motif at target promoter region (red bars only in IGV). The other TF, NFE2 cooperates with USF2 in an AND logical relation via protein-protein interaction. (TIF) [file pcbi.1004132.s002.tif]

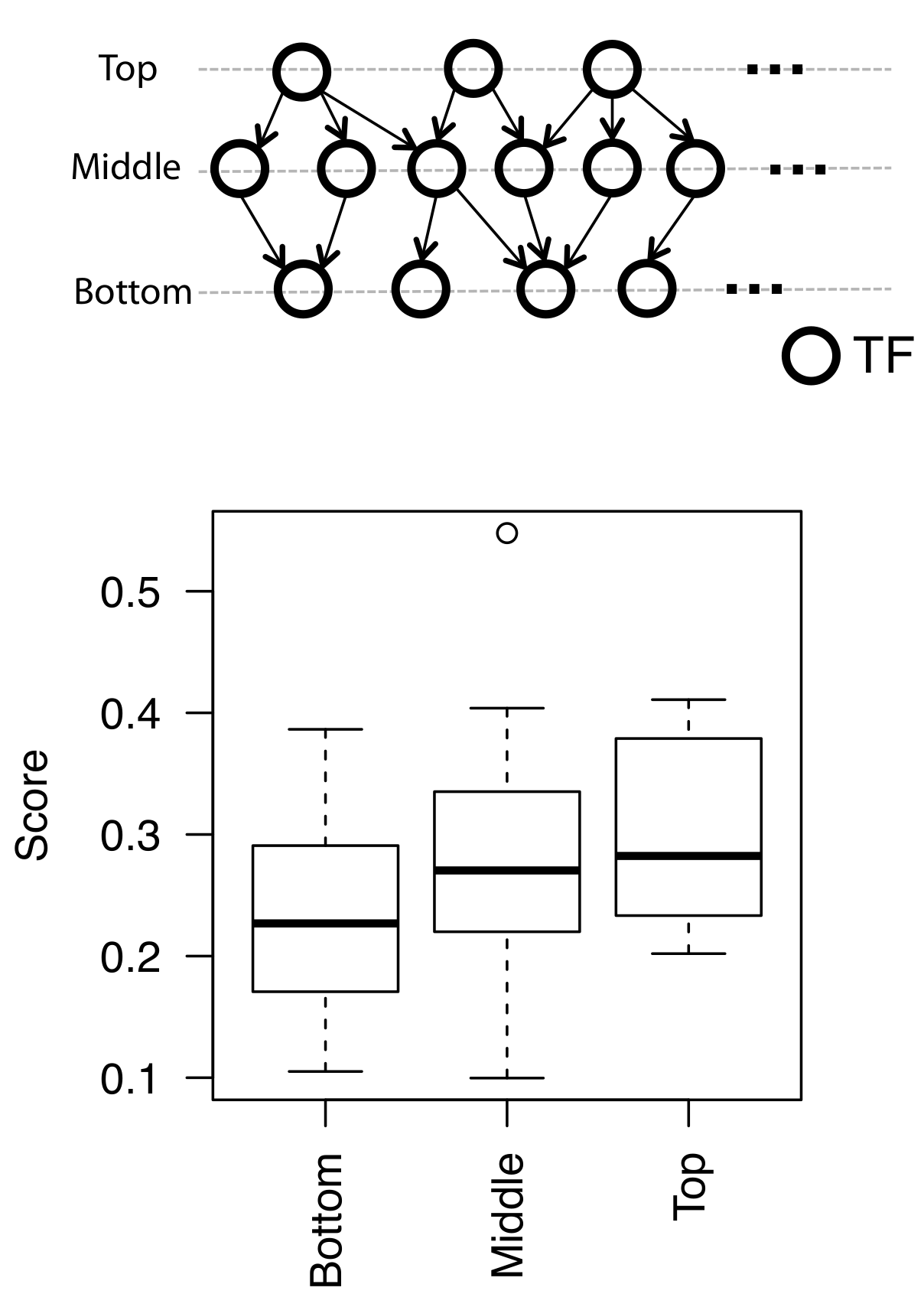

Supplement: S3 Fig — Boxplot displays the score distributions of the logic-gate-consistent triplets with targets being TFs at three different hierarchical layers: top, middle and bottom. The TFs at bottom have lower scores than others in yeast. (TIF) [file pcbi.1004132.s003.tif]

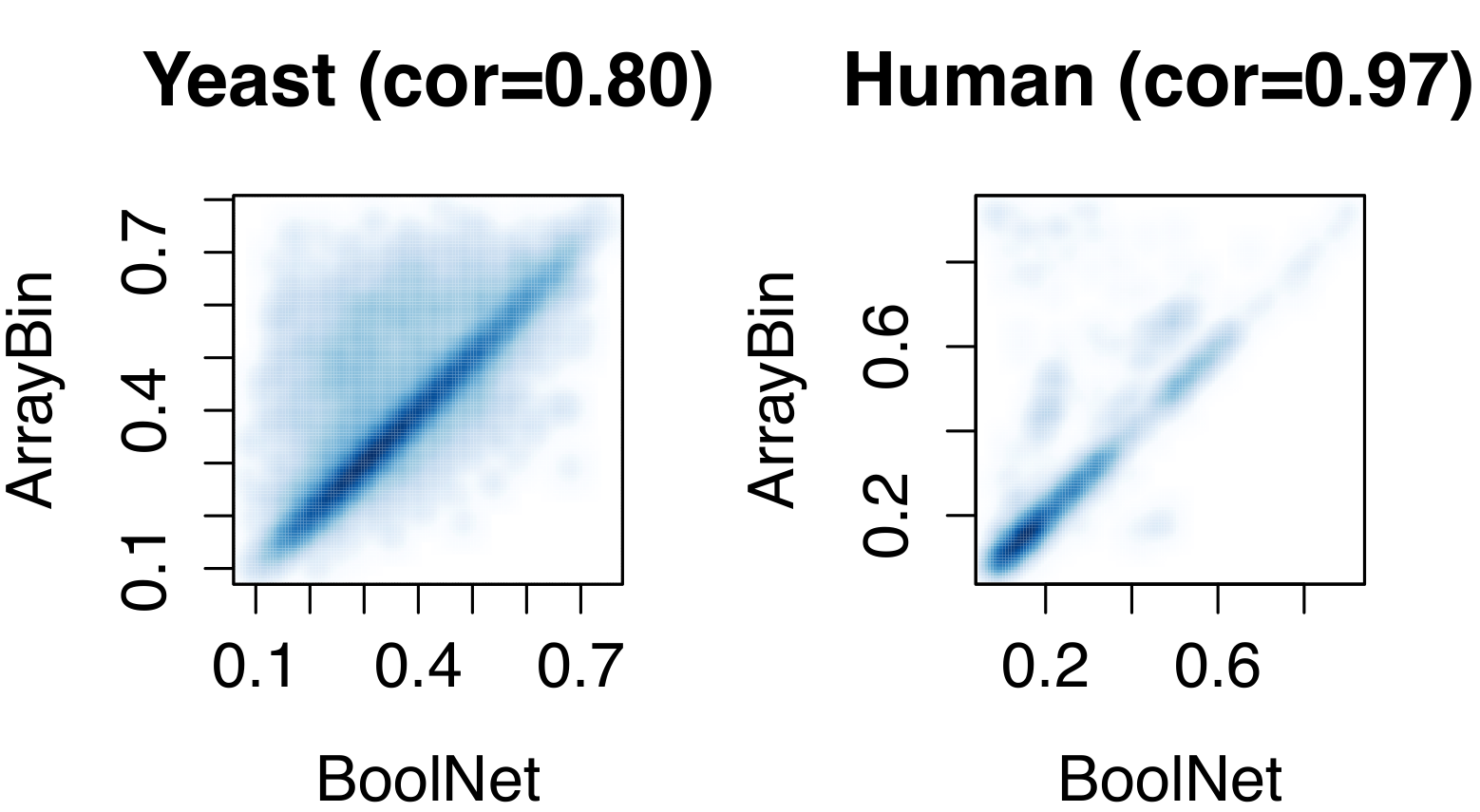

Supplement: S4 Fig — Scatterplots (left: yeast, right: human) display the consistency scores of logic-gate-consistent triplets that best match the same logic gates by Loregic using two binarized datasets: one is from the BoolNet method in this paper (x-axis), and another is from the ArrayBin method (y-axis) [45]. The scores are highly correlated between two methods (correlation = 0.80 in Yeast, and 0.97 in Human). (TIF) [file pcbi.1004132.s004.tif]
